# COVID-19 Outcome Relates With Circulating BDNF, According to Patient Adiposity and Age

**DOI:** 10.3389/fnut.2021.784429

**Published:** 2021-12-10

**Authors:** Luciele Guerra Minuzzi, Marília Seelaender, Bruna Spolador De Alencar Silva, Eduardo del Bosco Brunetti Cunha, Marina De Castro Deus, Franciane Thais Falcão Vasconcellos, Luis Felipe Beltrão Marqueze, Ana Carolina Gadotti, Cristina Pellegrino Baena, Telmo Pereira, Karsten Krüger, Andréa Novais Moreno Amaral, Ricardo Aurino Pinho, Fábio Santos Lira

**Affiliations:** ^1^Exercise and Immunometabolism Research Group, Postgraduation Program in Movement Sciences, Department of Physical Education, Universidade Estadual Paulista (UNESP), Presidente Prudente, Brazil; ^2^Cancer Metabolism Research Group, LIM26-HC, FMUSP, University of São Paulo, São Paulo, Brazil; ^3^Graduate Program in Health Sciences, School of Medicine, Pontificia Universidade Catolica do Parana, Curitiba, Brazil; ^4^Polytechnic Institute of Coimbra, Coimbra Health School, Coimbra, Portugal; ^5^Laboratory for Applied Health Research (LabinSaúde), Coimbra, Portugal; ^6^Department of Exercise Physiology and Sports Therapy, Institute of Sports Science, Justus-Liebig-University Giessen, Giessen, Germany

**Keywords:** adiponectin, leptin, BDNF, COVID-19 severity, adipose tissue hormones

## Abstract

**Background and Aims:** We evaluated adipose tissue-derived hormones, body composition, serum metabolic profile, levels of brain-derived neurotrophic factor (BDNF), and the association of these parameters with the clinical outcome in patients with COVID-19. We sought to examine whether obesity, sex, and age influence the adipose tissue endocrine response to the disease.

**Methods:** This prospective study investigated 145 hospitalized patients with COVID-19. Patients were categorized based on their body mass index (BMI), sex and age, and were also classified regarding their outcome after hospitalization as: (a) Non-ICU: patients hospitalized who did not receive intensive care; (b) ICU-survivor: patients admitted to the intensive care unit and discharged; (c) ICU-death: patients who died. Blood samples were collected by the hospital staff between the first and third day of hospitalization. Serum leptin, adiponectin and BDNF concentrations, triglycerides, total cholesterol and cholesterol fractions were performed following the manufacturer's guidelines.

**Results:** We demonstrate that BDNF levels predict intensive care (IC) need (*p* < 0.01). This association was found to be stronger in patients >60y (*p* = 0.026). Neither leptin nor adiponectin concentration was associated with IC requirement or with patient's outcome, while the BDNF/adiponectin ratio was closely associated with worsened outcomes (*p* < 0.01). BDNF concentration was similar between sexes, however tended to be lower in male patients (*p* = 0.023). In older patients, BDNF concentration was lower than that of younger patients (*p* = 0.020). These age and sex-specific differences should be considered when employing these potential markers for prognosis assessment. While appetite and body composition regulating hormones secreted by the white adipose tissue are not reliable predictors of disease severity, the ratio BDNF/adiponectin was indicative of patient status.

**Conclusion:** Thus, we propose that serum BDNF content and BDNF/adiponectin ratio may serve as tools predicting worsened prognosis in COVID-19, especially for male patients.

## Introduction

According to the WHO report of April 27th, 2021, the total number of confirmed patients with COVID-19 has risen sharply to 148,797,483, and 3,138,200 (2.1%) deaths were reported. A conspicuously higher mortality rate in overweight/obese patients is observed (1). It seems true, that higher adiposity is closely related to the aggravation of covid symptoms (2), and that the white adipose tissue (WAT) plays an active role in the response to the infection by SARS-CoV-2. The WAT is involved in the pathophysiology of various diseases (3–5), and by secreting a myriad of factors, it exerts influence on different compartments of the body, modulating an ample specter of physiological and pathological responses. Of particular interest is the capacity of WAT to actively express and secrete hormones and inflammatory factors. The likely contribution of the adipose tissue for covid-19-related “cytokine storm,” and hence, to the aggravated form of the disease and worsened prognosis, has been recently proposed (6–8).

Dugail et al. (2020) (9) and others (8, 10) have postulated that the infection of the adipose tissue by SARS-CoV-2 *via* angiotensin-converting enzyme 2 (ACE2) receptor expression and transmembrane protease serine 2 (TMPRSS2) might be involved with virus replication in WAT, increased lipolysis (releasing fatty acids into the circulation, including the inflammation-inducing saturated fatty acids), production and secretion of lipid active metabolites, cytokines, and dysregulation of WAT endocrine function. Indeed, individuals with higher adiposity and diabetes show augmented vulnerability to infectious diseases, especially those of the respiratory tract (11–13). Furthermore, the severity of various infectious respiratory diseases caused by viruses is increased in obese patients (14, 15).

Growing attention has been given to the inflammatory (mainly cytokine actions) contribution of WAT to the severity of COVID-19, while little (on 03/24/20, only 5 results could be retrieved in PubMed under the entry: adipose tissue hormone AND covid) information is available regarding the appetite and body composition regulating hormones secreted by the tissue. The hormones secreted by WAT, leptin, and adiponectin have, however, an important role in the regulation of the immune system (16, 17), which could be of relevance for patient outcome in covid-19, as hypothesized by De Bandt and Monin (18). Immune cells present leptin receptors, which, upon binding of the hormone, elicit changes in intracellular metabolism, translating the information about the nutritional status of the organism (19). Leptin receptors of immune cells have also been linked to the extent of the respiratory tract response to inflammatory factors such as INF-gamma, to virus clearance capacity and survival upon infection (20).

Adiponectin has been very recently (21) found to be associated with the regulation of lipid metabolism and inflammatory factor secretion in patients with SARS-CoV-2. This adipokine may thus influence both in direct and indirect form COVID-19 severity. Bearing in mind that adiponectin and leptin secretion is markedly altered in obesity (22), it seems plausible to put forth the proposition that these hormones may present a role in the aggravation of COVID-19 in overweight patients.

However, actual data on the association of these hormones and COVID-19 pathophysiology are missing in the literature. Another potential effect of leptin is regulating brain-derived neurotrophic factor (BDNF) presence and actions in WAT (23). BDNF is an agent that regulates lipolysis and many other WAT functions, by warranting the integrity and optimal function of sympathetic activity in the tissue. BDNF is known to be expressed by visceral adipose tissue in mammals (24). We sought therefore, to examine in COVID-19 patients with different severity of the disease, the adiponectin/leptin ratio as a predictor of outcome and the association of these hormones with BDNF concentration in the circulation.

## Materials and Methods

### Study Design

One hundred forty-five hospitalized patients diagnosed with COVID-19 admitted at the Marcelino Champagnat Hospital, Curitiba, Paraná, Brazil, between June and November 2020, were included in a prospective study. Patients were admitted only after signing the fully informed consent. The study was approved by the research ethics committee approval (number 31558020.8.0000.0103).

### Inclusion and Exclusion Criteria

Patients of both sexes were included when meeting the following criteria: being over 18 years old and showing clinical symptoms and positive RT-PCR for COVID-19. Sars-CoV-2 infection was confirmed by the clinical-radiological exam and nasopharyngeal swab polymerase chain reaction (PCR). Patients diagnosed with other viral infections or other common respiratory viruses were excluded as were those who underwent solid organ or hematological transplantation in the past.

### Patient Groups

In this study, we included 145 patients with COVID-19. Patients were categorized based on their body mass index (BMI) as: Non-obese = BMI < 29.9 kg/m^2^ (n = 78); Obese = BMI >30 kg/m^2^ (n = 67); Sex: Female (n = 59) and male (n = 86); Age: < 60 years old (n = 93) and ≥60 years old (n = 52). BMI was calculated based on the following formula: bodyweight in kilograms divided height in meters squared. COVID-19 patients were also classified regarding their outcome after hospitalization as: (a) Non-ICU: patients hospitalized who did not receive intensive care; (b) ICU-survivor: patients admitted to the intensive care unit and discharged; (c) ICU-death: patients who died. The statistical power of the sample size was calculated after sample analysis based on the linear regression between BDNF and outcome (death) observed in the study (*r*^2^ = 0.09, ρ^2^ = 0.101) and 95% confidence level using G^*^Power version 3.1.9.6. The power of the sample size of this study is 0.915.

### Patient Clinical Data and Blood Collection

The medical records of the patients provided the data on sex, age, body weight, height, coexisting diseases, clinical symptoms, peripheral oxygen saturation, continuous medication, and COVID-19-specific medication assessment, as well as the length of the hospital (ICU or ward) stay. Non-fasting blood samples (10 mL) were collected by the hospital staff between the first and third day of hospitalization. Peripheral blood was collected in vacutainer tubes without additives, containing separating gel, and kept at room temperature for 30 min to induce clotting. Samples were then centrifuged at 1500 rpm for 10 min at 4°C. The tubes remained at rest for 60 min in a vertical position and the serum was aliquoted and stored at −70°C until the biochemical tests were performed. Hemolyzed serum samples were discarded.

### Blood Parameters

The tubes were refrigerated for 1 h before centrifugation for 15 min at 3,000 rpm at 4°C (Centrifuge 5430 R, Eppendorf, Hamburg, Germany) for serum separation. Next, the serum content was stored at −20°C until frozen and stored at −80°C until analysis. Serum leptin, adiponectin and BDNF concentrations were assessed employing enzyme-linked immunosorbent assay (ELISA) (R&D System, Minneapolis, MN, USA). The assays were performed following the manufacturer's guidelines. Triglycerides (TAG), total cholesterol and cholesterol fractions (TC, HDL-c) were analyzed with commercial colorimetric kits (Labtest, Brazil) and Non-HDL cholesterol was calculated by subtracting HDL-c concentration from Triacylglycerol. The concentration of insulin and cortisol were analyzed with ELISA commercial kits (Monobind Inc., USA) and glucose content was assessed with a colorimetric kit (Labtest®, Brazil). The homeostatic model assessment of insulin resistance (HOMA-IR) index was calculated according to the formula: (glucose [mmol/L] × insulin [μIU/mL]/22.5) (25).

### Statistical Analysis

Data normality was tested using the Shapiro-Wilk test. Continuous values were expressed as medians and interquartile range (IQR) and categorical variables as counts and percentages. The comparisons among groups (non-obese vs. obese; female vs. male; < 60 years vs. ≥60 years) were performed with Mann-Whitney for non-parametric continuous variables. Categorical variables were compared using the Chi-square test. For the values of adipose-derived hormones and BDNF levels for patients with COVID-19 regarding their clinical outcome, data are mean ± Standard Error of the Mean. To compare the values from patients that were hospitalized without intensive care (Non-ICU), patients admitted to the intensive care unit and discharged (ICU-survivor), and patients who died (ICU-death), ANOVA was performed, followed by the Tukey test (normal distribution). For non-parametric data, Kruskal-Wallis test was performed, followed by the Dunn test. Linear regression was used to examine the associated factors. Then, binary logistic regression analysis was employed to identify factors associated with ICU admission and outcome—survivor or death. Univariate and multivariate binary logistic regression were performed to test the association between the dependent and the independent variables. The statistical significance was set at *p* < 0.05.

## Results

### Characteristics of the Study Cohort

Characteristics of the total of 145 hospitalized patients with confirmed COVID-19 at the Marcelino Champagnat Hospital, Curitiba, Paraná, Brazil are illustrated in (Table 1). Among the patients, 86 (59.3%) were male, and the median age was 53 (IQR 43–68) years. Patients were classified as obese or non-obese, considering BMI clusters = non-obese <29.9 kg/m^2^; obese >30 kg/m^2^. A total of 67 (46.2%) of these patients were obese. The median BMI in the Obese group and the Non-obese group were 32.9 (IQR 31–35) and 26.3 (IQR 24–27) kg/m^2^ (*p* < 0.001), respectively. There were no significant differences between the two subgroups in respect to sex (*p* = 0.669), age (*p* = 0.481), and use of medication (*p* = 0.120). Obese group showed a higher frequency of comorbidities (76.1%), when compared to the non-obese group (57.7%), *p* = 0.019. There were additionally, significant differences between these two subgroups in regard to diseases, as the former (obese group) showed a higher incidence of hypertension (*p* = 0.001), and dyslipidemia (*p* = 0.017) (Table 1).

**Table 1:** Characteristics of patients with Covid-19.

	**All patients**	**Non-Obese**	**Obese**	**P value**	**Non-ICU**	**ICU-survivor**	**ICU-death**	**P value**
N (%)	145 (100)	78 (53.8)	67 (46.2)		89 (61.4)	39 (26.9)	17 (11.7)	
Age, years	53 (43–68)	54 (42–68)	56 (43–69)	0.976	53 (43–65)^c^	58 (42–69)^c^	72 (50–78)	<0.0001
Body weight, kg	85 (73–98)	75 (69–85)	98 (88–108)	<0.001	83 (71–90)	90 (72–108)	90 (71–104)	0.145
BMI, kg·m^2^	29.4 (26–32)	26.3 (24–27)	32.9 (31–35)	<0.001	28.3 (25–30)	31.5 (27–34)^a^	31.9 (26–34)	0.001
**Sex**								
Female, *n* (%):	59 (40.7)	33 (42.3)	26 (38.8)	0.669	38 (42.7)	16 (41.0)	5 (29.4)	0.593
Male, *n* (%):	86 (59.3)	45 (57.7)	41 (61.2)		51 (57.3)	23 (59.0)	12 (70.6)	
**Age range**								
Age, <60 years, *n* (%):	93 (64.1)	48 (61.5)	45 (67.2)	0.481	63 (70.8)	27 (69.2)	3 (17.6)	<0.0001
Age, ≥60 years, *n* (%)	52 (35.9)	30 (38.5)	22 (32.8)		26 (29.2)	12 (30.8)	14 (82.4)	
**Comorbidities**								
No, *n* (%):	49 (33.8)	33 (42.3)	16 (23.9)	0.019	37 (41.6)	11 (28.2)	1 (5.9)	0.012
Yes, *n* (%):	96 (66.2)	45 (57.7)	51 (76.1)		52 (58.4)	28 (71.8)	16 (94.1)	
Hypertension, *n* (%):	53 (36.6)	19 (24.4)	34 (50.7)	0.001	25 (28.1)	17 (43.6)	11 (64.7)	0.009
Diabetes, *n* (%):	33 (22.8)	16 (20.5)	17 (25.4)	0.553	16 (18.0)	11 (28.2)	6 (35.3)	0.189
Dyslipidemia, *n* (%):	21 (14.5)	6 (7.7)	15 (22.4)	0.017	10 (11.2)	6 (15.4)	5 (29.4)	0.146
Respiratory diseases„ *n* (%):	13 (9.0)	5 (6.4)	8 (11.9)	0.383	5 (5.65)	6 (15.4)	2 (11.8)	0.187
Other, *n* (%):	66 (45.5)	34 (43.6)	32 (47.8)	0.621	34 (38.2)	19 (48.7)	13 (76.5)	0.013
**Medication**								
No, *n* (%):	53 (36.6)	33 (42.3)	20 (29.9)	0.120	36 (40.4)	16 (41.0)	1 (5.9)	0.020
Yes, *n* (%)	92 (63.4)	45 (57.7)	47 (70.1)		53 (59.6)	23 (59.0)	16 (94.1)	

*Values are reported as a number (%) or median and interquartile range (Q1–Q3). Mean difference: Chi-square (Categorical variables) and Mann-Whitney test (Continuous variables). Body mass index classes: non-obese <29.9 kg·m^2^; obese > 30 kg·m^2^*.

We further divided the patients according to the requirement or not of intensive care (ICU). A total of 56 patients (38.6%) were admitted to the ICU, followed by discharge (26.9%, ICU-survivor subgroup) or death (11.7%, ICU-death subgroup). COVID-19 patients receiving intensive care were on average older (58.5, IQR 46–73 years), when compared to the Non-intensive care group (50, IQR 40–63 years) (*p* = 0.006) (Table 1).

Patients who needed intensive care were more frequently obese (64.3%, *p* < 0.001) and presented more comorbidities (78.6 %, *p* = 0.013). The mean BMI of critically ill patients admitted to ICU was around 31.5 kg/m^2^ at admission. In fact, in our sample, 75% of all SARS-CoV-2 positive patients that required IC had a BMI of 30 kg/m^2^ or higher (mean 31.8 kg/m^2^). The other 25% had a mean BMI of 26.9 kg/m^2^. Among the non-survivors, 94.1% were under chronic medication for comorbidities, mainly for arterial hypertension (64.7%) or multiple diseases (76.5%) (Table 1).

### Biochemical and Metabolic Profiles

The baseline biochemical and metabolic profile results of all patients and the comparison between the subgroups stratified within the criteria of obesity and need for intensive care are presented in Table 2. The patients from the Obese group showed higher concentration of TAG, non-HDL cholesterol and cortisol, compared to the Non-obese patients (*p* < 0.05). In the patients who needed intensive care, triglyceride levels were also higher (*p* = 0.001). Thus, we observed that the patients who went to the ICU showed higher TAG, compared to the non-ICU group; while we failed to find differences in total cholesterol content among groups. HDL-c was higher in female, when compared to male patients (*p* = 0.027, 29.8 (IQR 25–35) and 27.1 (IQR 24–32), respectively) (Supplementary Table 1).

**Table 2:** Biochemical and metabolic profiles of patients with COVID-19.

	**All Patients (*n* = 145)**	**Non-Obese** **(*n* = 78)**	**Obese (*n* = 67)**	**P value**	**Non-ICU (*n* = 89)**	**ICU-survivor** **(*N* = 39)**	**ICU-Death (*N* = 17)**	**P value**
Cholesterol, mg·dL^−1^	184.2 (156–216)	182.0 (157–206)	188.6 (154–226)	0.157	183.6 (157–209)	200.5 (156–232)	183.8 (155–212)	0.435
Triglyceride, mg·dL^−1^	136.2 (111–185)	125.4 (106–156)	177.0 (121–223)	<0.001	130.7 (110–175)^b^	181.2 (131–211)	125.0 (102–137)^b^	0.001
HDL-c, mg·dL^−1^	28.1 (24–33)	28.3 (24–33)	28.0 (24–33)	0.818	28.5 (25–35)	27.2 (24–32)	25.6 (20–33)	0.137
Non-HDL-c, mg·dL^−1^	153.4 (125–186)	147.4 (122–178)	160.5 (126–195)	0.140	148.0 (123–177)	175.1 (125–201)	160.5 (129–185)	0.217
Insulin, μU·mL^−1^	32.4 (20–46)	32.3 (22–45)	34.5 (18–48)	0.530	34.3 (21–46)	35.0 (19–47)	25.4 (15–44)	0.551
Glucose, mg·dL^−1^	90.9 (79–109)	88.5 (80–106)	96.5 (79–113)	0.295	88.8 (80–103)	98.2 (79–120)	97.5 (83–111)	0.187
HOMA-IR	7.7 (4–12)	7.2 (5–12)	9.1 (4–12)	0.986	7.3 (4–12)	9.4 (5–13)	6.2 (3–11)	0.417
Cortisol nmol·L^−1^	6.6 (2–16)	5.7 (2–10)	10.2 (6–49)	<0.001	6.7 (3–12)	8.3 (3–48)	48.9 (4–50)	0.065

*Values are presented as median and interquartile range (Q1–Q3). Mean difference: Mann-Whitney test*.

*Intensive care contained discharged and dead patients*.

Only 11.2% of the COVID-19 patients did not present insulin resistance, as indicated by the index based on fasting blood insulin and glucose concentrations (HOMA-IR) (Table 2). Although there was no statistical difference in HOMA-IR between Obese and non-obese groups, as all mean values were quite high (median 8.4), when we compared these with normal values (under 2.5).

### Adipose-Derived Hormones Concentration in Patients With COVID-19 in the Different Subgroups

We analyzed and compared the results for adiponectin, leptin, and the adiponectin/leptin ratio, to understand the endocrine physiology of adipose tissue response in COVID-19, considering the effect of the factors: obesity, sex, and age.

#### BMI

**Figure 1:**
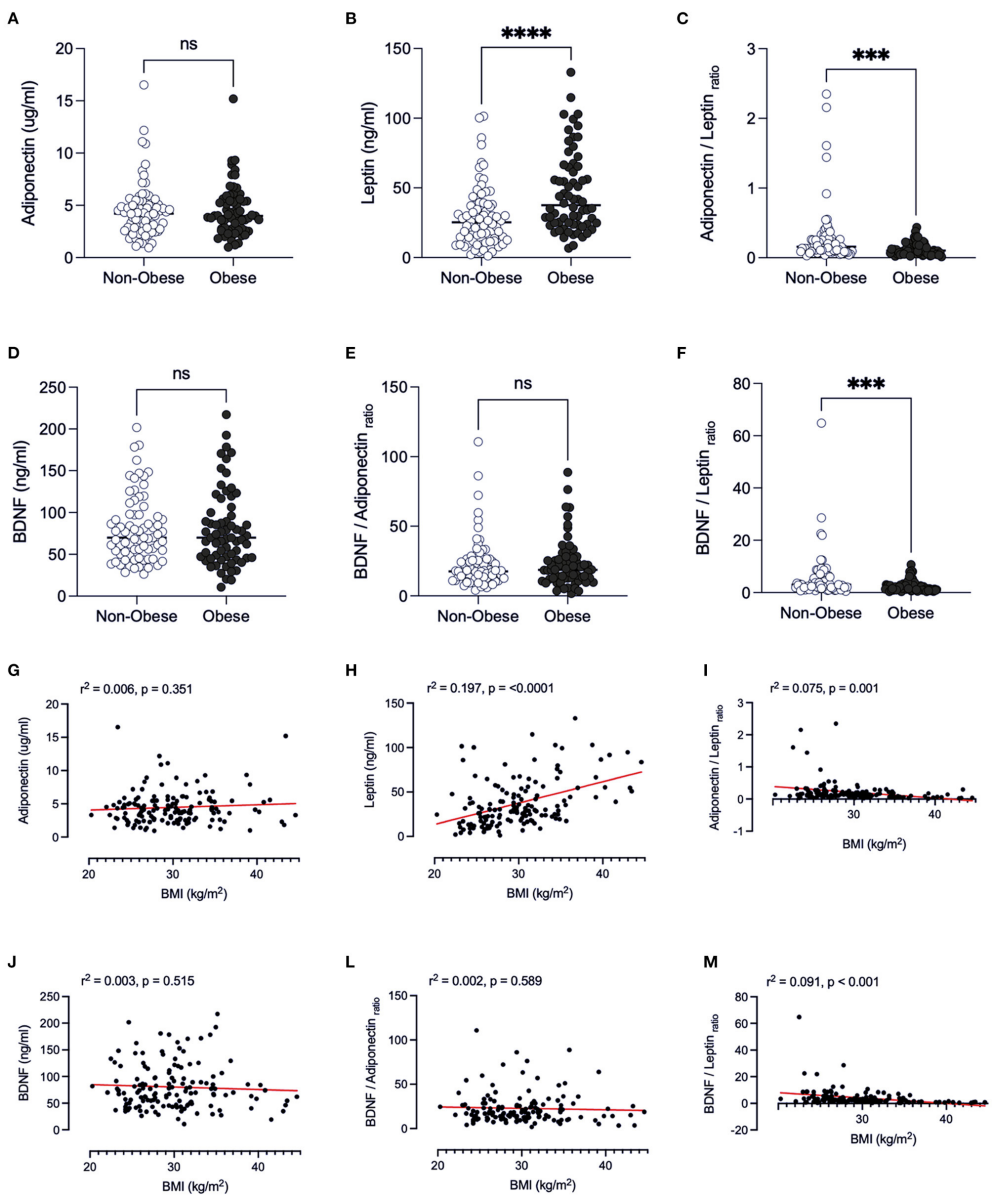
Comparison of adipose-derived hormones levels between non-obese and obese patients with Covid-19. Values expressed as median of adiponectin (ug/ml) **(A)**, leptin (ng/ml) **(B)**, adiponectin/leptin ratio **(C)**, BDNF (ng/ml) **(D)**, BDNF/Adiponectin ratio **(E)**, BDNF/Leptin ratio **(F)**, BDNF / Adiponectin/Leptin ratio **(G)** were compared between two groups. Dashed box presents Linear regression curve of adiponectin **(G)**, leptin **(H)**, adiponectin/leptin ratio **(I)**; BDNF **(J)**, BDNF/Adiponectin ratio **(L)**, BDNF/Leptin ratio **(M)** as dependent variable (Y axis) with body index mass (BMI) as independent variable (X axis). Significance: ***p < 0.001; ****p < 0.0001.

Obesity caused leptin to be higher when compared to non-obese individuals (*p* < 0.001) (Figure 1B). The leptin values may be associated with the lower adiponectin/leptin ratio found in the obese individuals in the study (0.159, IQR = 0.09–0.27), when compared to non-obese patients (*p* < 0.001, Figure 1C), as the adiponectin levels are similar (Figure 1A). Adiponectin/leptin ratio is an important clinical parameter that reflects the functionality of the adipose tissue and may be employed to identify those subjects more susceptible to developing cardiometabolic diseases (ratio > 1.0 = normal, between 0.5 and 1.0 = moderate-medium increased risk, <0.5 = severe increase in cardiometabolic risk) (22). In this regard, we analyzed the profile of the adipose tissue-secreted hormones and the adiponectin/leptin ratio, as the BMI increases, by linear regression analyses (Figures 1G–I). With the increment of BMI (suggested increased adiposity), the level of leptin increases, as expected (*r*^2^ = 0.197; *p* < 0.0001, Figure 1H). We also found that the adiponectin/leptin ratio decreases as BMI increases, and from 30 kg/m^2^ the ratio was below 0.4 (suggesting cardiometabolic risk) (*r*^2^ = 0.075, *p* < 0.01, Figure 1I).

There was no significant difference in BDNF levels between the obese and non-obese groups (*p* = 0.847) (Figure 1D). In the present study, the ratio of BDNF to leptin was significantly lower in obese individuals than non-obese (*p* = 0.0001, Figure 1F), whilst the BDNF/adiponectin ratio was similar (Figure 1E). This finding was reinforced by the demonstration that there is a decrease in this ratio as BMI increases (Figure 1M) which can be pointed out as an indicator of endocrine physiology of the adipose tissue depots.

#### Sex

**Figure 2:**
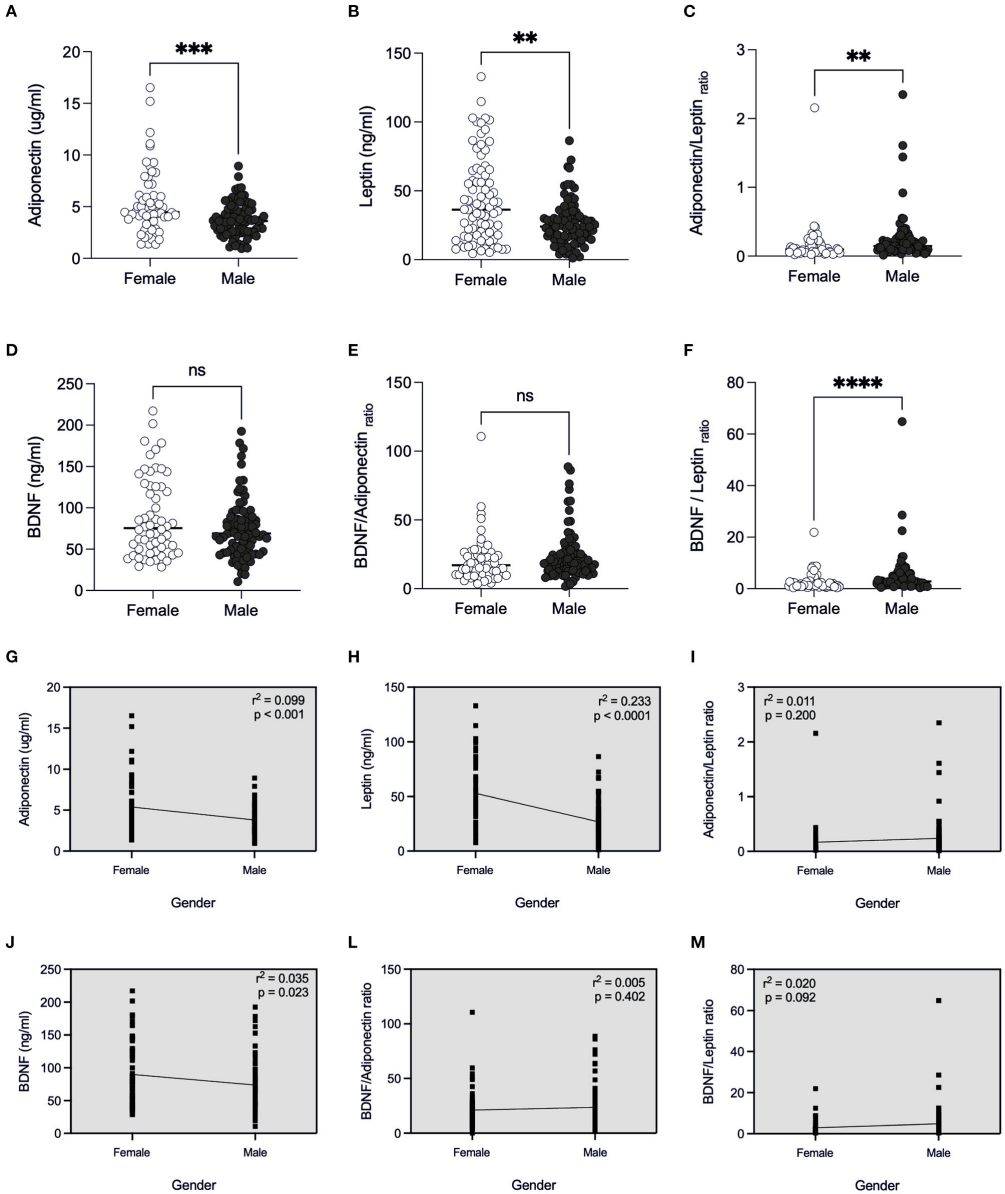
Comparison of adipose-derived hormones levels between female and male patients with Covid-19. Values expressed as median of adiponectin (ug/ml) **(A)**, leptin (ng/ml) **(B)**, adiponectin/leptin ratio **(C)**, BDNF (ng/ml) **(D)**, BDNF/Adiponectin ratio **(E)**, BDNF/Leptin ratio **(F)**, BDNF / Adiponectin/Leptin ratio **(G)** were compared between two groups. Dashed box presents linear regression curve of adiponectin **(G)**, leptin **(H)**, adiponectin/leptin ratio **(I)**; BDNF **(J)**, BDNF/Adiponectin ratio **(L)**, BDNF/Leptin ratio **(M)** as dependent variable (Y axis) with sex (female or male) as independent variable (X axis). Significance: **p < 0.01; ***p < 0.001; ****p < 0.0001.

By examining the factor sex (Figures 2A–M), we found that male patients presented lower concentration of adiponectin [3.60 (IQR 2.57–4.52)] (Figure 2A), and of leptin [24.07 (IQR 14.76–33.25)] (Figure 2B) and therefore, higher adiponectin/leptin ratio [0.14 (IQR 0.09–0.24)] (Figure 2C), when compared to female counterparts (*p* < 0.05) (4.47 (IQR 3.58–6.10), 48.67 (IQR 28.52–77.81) and 0.09 (IQR 0.05–0.19), respectively). BDNF content was similar between sexes (Figure 2D), however linear regression showed that BDNF concentration was lower in male patients (*r*^2^ = 0.035, *p* = 0.023, Figure 2J).

#### Age

**Figure 3:**
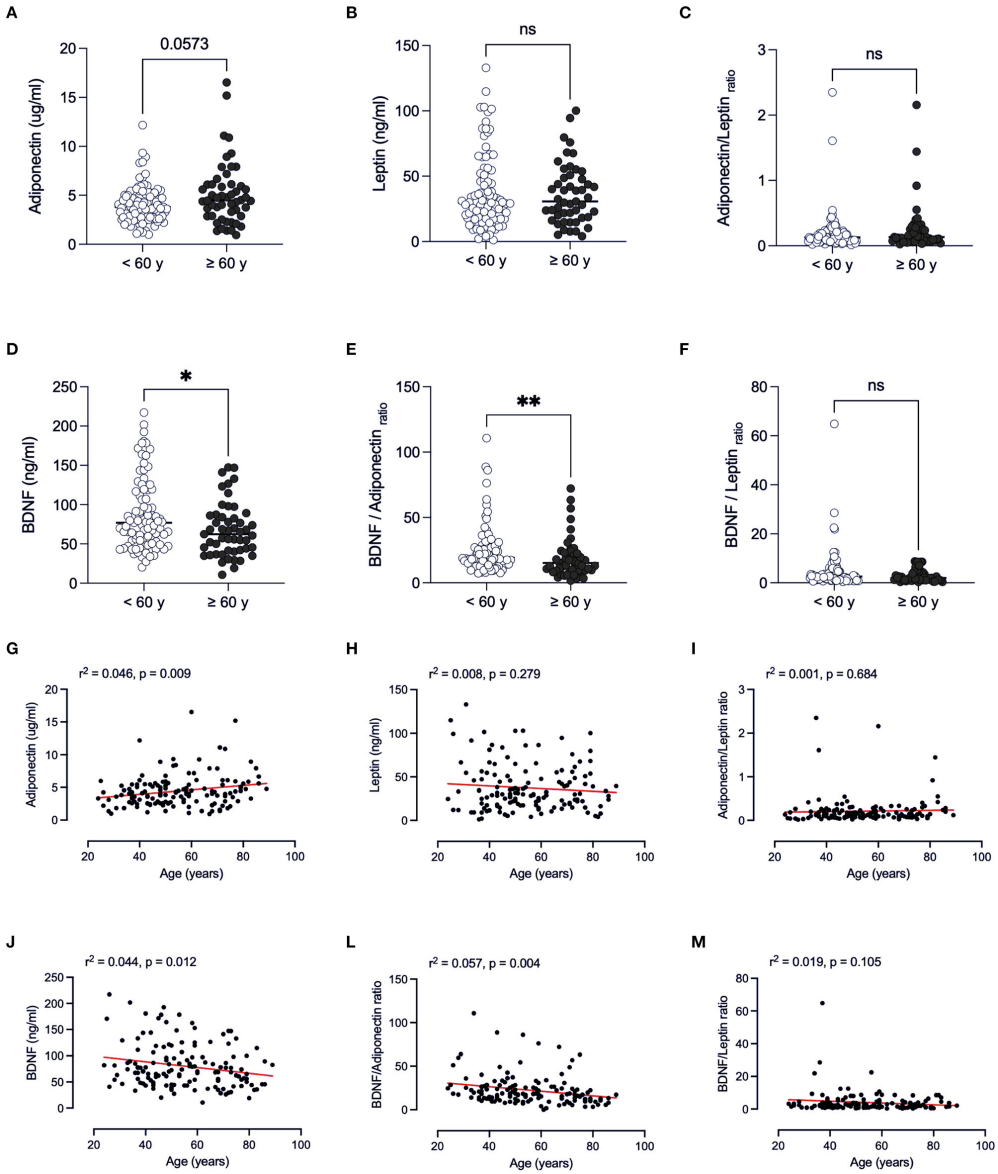
Comparison of adipose-derived hormone levels between age ranges (<60 years vs. ≥60 years) of patients with Covid-19. Values expressed as median of adiponectin (ug/ml) **(A)**, leptin (ng/ml) **(B)**, adiponectin/leptin ratio **(C)**, BDNF (ng/ml) **(D)**, BDNF/Adiponectin ratio **(E)**, BDNF/Leptin ratio **(F)**, BDNF / Adiponectin/Leptin ratio **(G)** were compared between two groups. Dashed box presents linear regression curve of adiponectin **(G)**, leptin **(H)**, adiponectin/leptin ratio **(I)**; BDNF **(J)**, BDNF/Adiponectin ratio **(L)**, BDNF/Leptin ratio **(M)** as dependent variable (Y axis) with age as independent variable (X axis). Significance: ^*^p < 0.05; **p < 0.01.

When considering the effect of age (Figures 3A–M), the values of the above-mentioned hormones were similar in individuals <60 years vs. ≥60 years (*p* > 0.05) (Figures 3A–C). In patients ≥60 years, BDNF concentrations were lower than those of younger patients <60 years old (*p* = 0.020) (Figure 3D). We examined whether adipose-derived tissue hormones and BDNF levels were related to age by a series of linear regression analyses. A significant effect of age was found for adiponectin, BDNF and all BNDF-calculated ratios. These findings suggest that an age-related decline in BDNF levels partially contributes to the adverse outcome with advancing age.

### Clinical Outcome

In the entire cohort of 145 patients with COVID-19, a total of 17 patients died (mortality 11.7%). Individuals who did not require ICU spent a shorter interval in the hospital (6.0 days (IQR 3–10), when compared to those who were under intensive care who were discharged [ICU-survivor, 18 days of hospitalization (IQR 13–28)] or who died (ICU-death, 21 days of hospitalization [IQR 13–25)] (*p* < 0.01) (Table 3). When dividing the patients according to sex, age, and obesity, the same was observed (Table 3). Intubation (36 patients, 24.8%) was a clear indicator of COVID-19 progression and severity in hospitalized patients, as expected (Table 3).

**Table 3:** Comparison of the Clinical Outcomes of Patients between the Non-obese vs. Obese, Female vs. Male, and by age range groups.

**Clinical outcome**	**Non-ICU**		**ICU-survivor**		**ICU-death**		
**Obese X non-obese**	**Non-obese** **(*n* = 58)**	**Obese** **(*n* = 31)**	**Non-obese** **(*n* = 14)**	**Obese** **(*n* = 25)**	**Non-obese** **(*n* = 6)**	**Obese** **(*n* = 11)**	***P* value**
Hospitalization time (days)	5 (3–10)	6 (3–10)	17.5 (12–24)^a^	18 (12–28)^a^	18 (13–22)^a^	22 (11–27)^a^	<0.0001
Intubation, *n* (%)			6 (42.8)	13 (52.0)	6 (100.0)	11 (100.0)	
In-hospital mortality, *n* (%)					6 (35.3)	11 (64.7)	
Female X Male	Female (*n* = 38)	Male (*n* = 51)	Female (*n* = 16)	Male (*n* = 23)	Female (*n* = 5)	Male (*n* = 12)	P value
Hospitalization time (days)	4.5 (3–10)	6 (4–10)	19.5 (13–27)^a^	17 (11–28)^a^	22 (14–27)^a^	20 (9–25)^a^	<0.0001
Intubation, *n* (%)			8 (50)	11 (47.8)	5 (100)	12 (100)	
In-hospital mortality, *n* (%)					5 (29.4)	12 (70.6)	
<60 y vs. >60 y	<60 y (*n* = 63)	≥60 y (*n* = 26)	<60 y (*n* = 27)	≥60 y (*n* = 12)	<60 y (*n* = 3)	≥60 y (*n* = 14)	P value
Hospitalization time (days)	5 (3–10)	6 (3–10)	17 (11–21) ^a^	25 (16–29)^a^	11 (>8)	21 (15–25)^a^	<0.0001
Intubation, n (%)			11 (40.7)	8 (66.7)	3 (100)	14 (100)	
In-hospital mortality, *n* (%)					3 (17.6)	14 (82.4)	

*Values are reported as a number (%) or median and interquartile range (Q1–Q3)*.

*Body mass index classes: non-obese <29.9 kg·m^2^; obese >30 kg·m^2^*.

*Kruskal-Wallis test for differences between clinical results within groups, with statistical significance represent by symbols*:

a *compared to Non-IC*.

**Figure 4:**
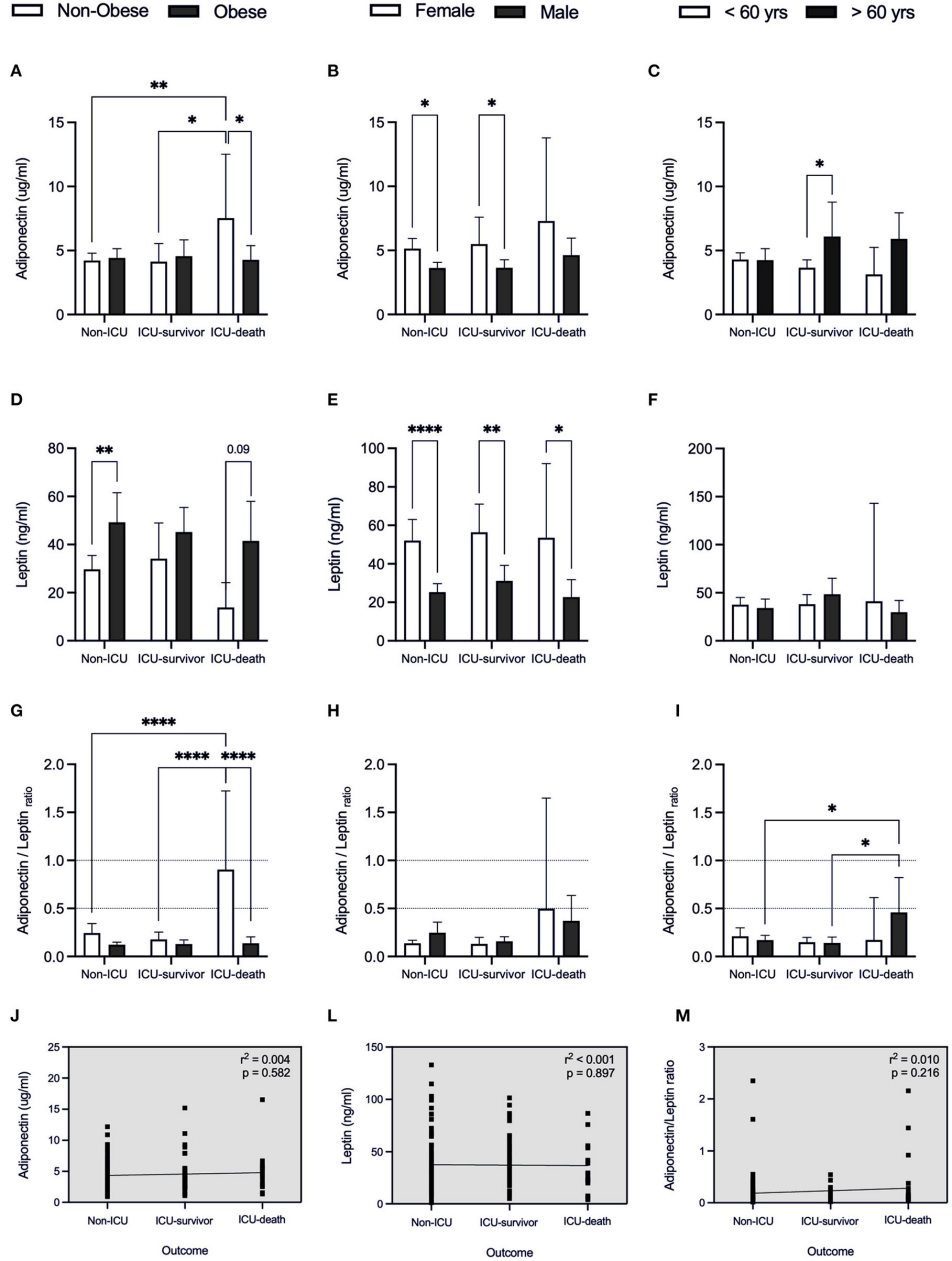
Adipose-derived hormones for patients with COVID-19 in relation to clinical outcome. Values expressed as mean with 95% Confidence Interval for patients that were hospitalized without intensive care (Non-ICU), patients admitted to the intensive care unit and discharged (ICU-survivor) and patients who died (ICU-death), including: adiponectin **(A–C)**, leptin **(D–F)**, and adiponectin/leptin ratio **(G–I)** related to body index mass (BMI), sex and age, respectively. Dashed box presents linear regression curve of adiponectin **(J)**, leptin **(L)**, and adiponectin/leptin ratio **(M)** as dependent variable (Y axis) with outcome as independent variable (X axis). Significance: *p < 0.05; **p < 0.01; ****p < 0.0001.

Regarding WAT-derived hormones (Figures 4A–M), neither leptin nor adiponectin levels were factors linked with admission to ICU, regardless of the outcome of obese patients (Figures 4A,D). Despite the finding that leptin plasma content was higher in obese individuals when compared to non-obese individuals (Figure 1B), only for the Non-ICU subgroup, this difference was significant (*p* = 0.016) (Figure 4D). A higher concentration of leptin was found in females compared to male patients (Figure 1H), independent of the type of hospitalization (Non-ICU *p* < 0.001; ICU-survivor *p* < 0.001 and ICU-death *p* = 0.048) (Figure 4E).

Curiously, in non-obese individuals who required ICU and died (7.7%), the adiponectin/leptin ratio was higher, when compared to non-obese individuals in the subgroups Non-ICU and ICU-survivor (*p* = 0.018, *p* = 0.008, respectively) (Figure 4G). This suggests the results be associated with the tendency for lower leptin concentration and higher adiponectin values in non-obese individuals who passed (*p* = 0.09; Figure 4D).

When we consider WAT-derived hormones according to sex and age (≥60 years and <60 years), no differences were detected when comparing the Non-ICU, ICU-survivor, and ICU-death subgroups (*p* < 0.05) (Figures 4B,C,E,F). Nevertheless, an increased adiponectin/leptin ratio was detected in patients ≥60 years who did not survive (Figure 4I).

**Figure 5:**
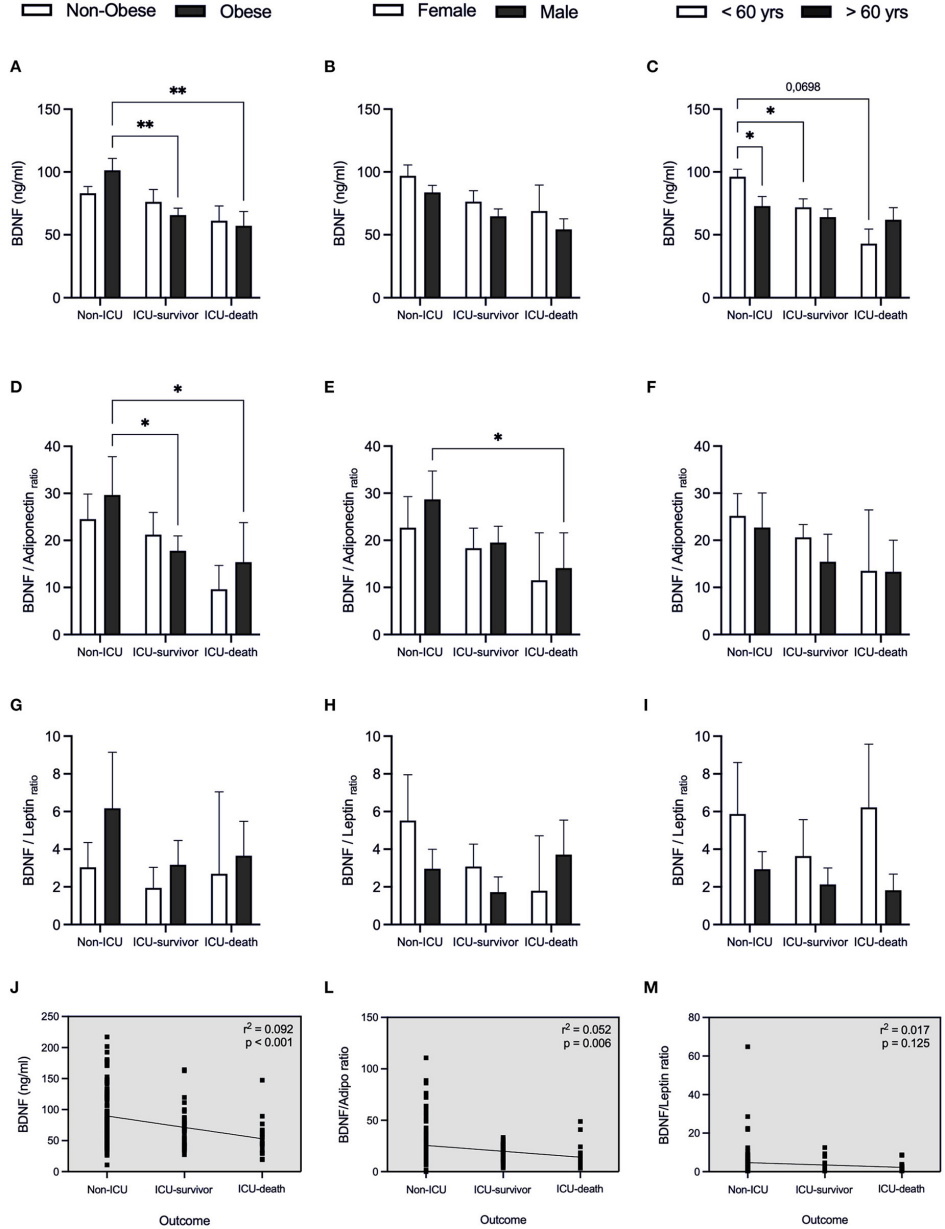
BDNF and BDNF-related ratios for patients with COVID-19 in relation to clinical outcome. Values expressed as mean with 95% Confidence Interval for patients that were hospitalized without intensive care (Non-ICU), patients admitted to the intensive care unit and discharged (ICU-survivor) and patients who died (ICU-death), including: BDNF **(A–C)**, BDNF/adiponectin ratio **(D–F)**, and BDNF/leptin ratio **(G–I)** related to body index mass (BMI), sex and age, respectively. Dashed box presents linear regression curve of adiponectin **(J)**, leptin **(L)**, and adiponectin/leptin ratio **(M)** as dependent variable (Y axis) with outcome as independent variable (X axis). Significance: *p < 0.05; **p < 0.01.

Considering BDNF concentration (Figures 5A–M), besides having found that BDNF levels are related to sex and age (Figures 2J, 3J), we also observed that in the obese individuals who did not require IC, these were higher, when compared with the group's ICU-survivor and ICU-death (*p* = 0.007; *p* = 0.009, respectively) (Figure 5A). The under 60 y patients who did not survive, showed a tendency to present lower BDNF levels than the non-ICU subgroup (*p* = 0.0698, Figure 5C). The ratio of BDNF/ adiponectin varies in parallel with different severities and outcomes (Figure 5L), pointing out that adipose endocrine function is important in COVID-19.

Linear regression revealed that higher BDNF levels are negatively correlated with disease severity, with BDNF values being lower in patients who died (*r*^2^ = 0.092, *p* < 0.001; Figure 5J). A beta coefficient of *b* = −18.34 was found for this correlation, indicating that for patients requiring IC (both survivor and death groups) we can expect a decrease in BDNF in 18.34 ng/ml each condition. This leads us to infer BDNF to be a possible marker of adverse clinical outcomes for COVID-19 patients.

In univariable logistic regression, Age, BMI, BDNF, BDNF/Adip ratio, hospitalization days, hypertension and respiratory diseases were significantly associated with ICU admission (all *p* < 0.05), whilst in-hospital death was significantly associated with age, BDNF, hospitalization days, hypertension and respiratory diseases, and ongoing pharmacological treatment (see Supplementary Table 2).

In multivariable regression analysis, designed to test the possibility of confounding relations of BDNF with other clinically relevant independent variables, BDNF hold a significant association with ICU admission (OR = 0.987; 95%IC:0.977 to 998; *p* = 0.018) in a model including Age (OR = 1.038, 95%IC: 1.011 to 1.065, *p* = 0.005), BMI (OR = 1.142, 95%IC: 1.058 to 1.232, *p* = 0.001), and Sex (0.779; 95%IC:0.352 to 1.727; *p* = 0.539). With Death as the dependent variable, BDNF lost the significant association (OR:0.985; 95%IC:0.966 to 1.005; *p* = 0.146) in a model also adjusted for Age, BMI and Sex, in which only Age maintained a significant association with Death (OR: 1.098; 95%IC: 1.048 to 1.151; *p* < 0.001). Similar results were depicted for the BDNF/Adip ratio, as portrayed in Supplementary Table 3. In additional multivariable logistic regression models, BDNF, BDNF/Adip ratio, hypertension and respiratory diseases were shown to be predictors of ICU admission, while hypertension and respiratory diseases were independent predictors of patients' risk of death (Supplementary Table 4). However, after adjusting for BDNF, BDNF/Adip ratio, Age, and BMI, logistic regression showed that hypertension and respiratory diseases are not predictors of death, being Age the only independent variable holding significant association with Death in either multivariable models considered (Supplementary Table 5).

## Discussion

In this prospective study, we examined adipose tissue-derived hormones involved in the control of appetite and body composition, the metabolic profile, and the levels of BDNF in the circulation, and the association of these parameters with disease severity and outcome in patients with COVID-19. The original findings show that (a) BDNF serum concentration is associated with disease outcome; (b) appetite and body composition regulating hormones secreted by WAT are not reliable predictors of disease severity, while the ratio BDNF/adiponectin was informative of patient status; (c) there are age and sex-specific changes that should be considered when employing these potential markers for prognosis assessment.

We report a linear and inverse relationship between BDNF and adverse outcomes in patients with COVID-19. The results also show that circulating BDNF declines with advancing age. In a COVID-19 condition, a hypothetical model suggested that the down-regulation of ACE-2 in the brain by SARS-CoV-2 inhibits the release of neurotropic factors such as BDNF (26). Recently, Azoulay and coll. (2020) showed that COVID-19 patients with moderate to severe disease showed decreased circulating content of BDNF when compared with those presenting milder symptoms (–~15%) (27). In this small study, the authors found that recovery from COVID-19 was associated with the augment of circulating BDNF (27). In the present study, 145 patients were examined, and we report a conspicuous association between BDNF levels and the severity of the disease, corroborating previous evidence that SARS-CoV-2 infection leads to down-regulation of the ACE2 receptors and this might be an aggravating factor for patients with medical conditions (28) and the suggestion that recovery from SARS-CoV-2 infection is associated with serum BDNF restauration (27).

BDNF plays a critical role in neuronal survival, growth, plasticity, and is essential for learning and memory (29). Wang and coworkers have demonstrated that ACE2 knockout mice exhibit a decrease in BDNF in the hippocampus region, and significant impairments of cognitive function compared with WT mice (30). In the same way, Angiotensin II type 1 receptor (AT1) blockade prevented memory impairment *via* up-regulation of BDNF (31). The infection of the SARS-CoV-2 in the brain increases the local levels of Angiotensin II by ACE2 down-regulation. It was suggested that the interaction of Angiotensin II with AT1 receptor increases kynurenine metabolites, producing pro-oxidative and pro-inflammatory effects, resulting in decreased levels of central BDNF (32). All these elements play a critical role in the impairment of cognitive function and a highly orchestrated immune-inflammatory response that ultimately could result in the development of depression (32). It is possible, therefore, that SARS-CoV-2 dampens BDNF synthesis and release and elicits neurologic symptoms (33). We show a clear association between COVID-19 prognosis and circulating BDNF, as patients with lower values consistently showed worsened outcome.

The involvement of BDNF in inflammatory conditions is complex (34). BDNF plays a role in the maturation and survival of T lymphocytes, especially under stress (35). Thus, in inflammatory conditions such as obesity and type 2 diabetes, immune cells (via increased IL-6) are stimulated to produce neurotrophins, like BDNF, to minimize the neuronal damage associated with obesity (36, 37). Consequently, it was suggested that a higher concentration of subunit pro-BDNF stimulates the infiltration of immune cells, culminating in an increase in the expression of proinflammatory cytokines (38). Thus, it is possible that in presence of viral infection and metabolic disturbance, as in COVID-19, the relationship between BDNF and inflammation is modified or even, impaired (seen by high BDNF levels, ~100 ng/ml in obese patients with COVID-19 when they were admitted to hospital). Different studies have shown either that circulating BDNF levels are higher in obese subjects than in controls (39, 40) or that there are no differences between the two groups (41). An upregulation of pro-BDNF expression in PDGFRα^+^ adipocyte progenitors was indicated as a feature of adipose tissue aging, suggesting that inhibition of BDNF expression in adipocyte progenitors could be potentially beneficial to prevent age-related adipose tissue dysfunction (42). However, in our study, we evaluated serum mature BDNF levels that have opposite effects of pro-BDNF (43). Interestingly, serum BDNF levels didn't differ across severity groups in patients >60yrs, while a reduction of BDNF in patients <60 yrs was observed with the progression of COVID-19 severity. This data reinforces the idea that increased mature BDNF isoform is related to the improvement of metabolic function, while the pro-BDNF isoform is related to the adipose tissue disfunction as observed by Song et al. (42). In addition, higher cortisol levels found in obese subjects and a tendency (*p* = 0.06) for higher levels of the same hormone in patients who died (ICU-death) suggest central hyperstimulation on the hypothalamus-adrenal-pituitary axis during SARS-CoV-2 infection. This scenario might favor a disconnection between central and peripheral communication.

BDNF is an important pleiotropic protein directly related to neuron and brain health, commonly inversely associated with obesity (34). In addition, the sympathetic activity in the adipose tissue can regulate lipolysis *via* BDNF (23). Emerging evidence links BNDF with SARS-CoV-2 infection dampens BDNF synthesis and release, which favors COVID-19 associated neurologic symptoms (33), however the metabolic role of BDNF in COVID-19 patients has been largely overlooked. Recently, an interplay between BDNF and adiponectin in the regulation of fat mass was proposed (44). Furthermore, it was suggested that adiponectin, selectively produced by the white adipose tissue, strongly correlates to the inflammatory process triggered by Sars-CoV-2 infection (21). Adiponectin concentration is inversely associated with the detection of cardiometabolic risk markers, and this hormone reflects adipose tissue physiological status (45). Our results show that adiponectin levels increased linearly with aging, and adiponectin serum content was also higher in non-obese patients who died and in older patients who required IC, which could reflect an attempt by the adipose tissue to counteract inflammation in COVID-19 (21). The BDNF/adiponectin ratio seems to be a reliable marker of the outcome.

Our data show that both leptin and adiponectin alone are not predicting factors for IC requirement or outcome in obese individuals. Conversely, the adiponectin/leptin ratio exhibited differences related with BMI (*r* = −0.274, *p* = 0.001), and thus this ratio may be employed as a tool for the assessment of obesity-associated cardiometabolic risk (22). Likewise, this ratio may be also informative of COVID-19 prognostic, as the obese patients with lower values are also the ones with the more severe form of the disease. A recent study from Italy showed that Adip/Lep ratio was associated with systemic inflammation in COVID-19 patients, where patients with moderate severity showed the highest Adip/Lep ratio values (46). In addition, the aforementioned study found that mortality tended to decrease with increasing Adip/Lep tertiles, suggesting an inadequate anti-inflammatory response in these patients (46). Conversely, we didn't find association between the Adip/Lep ratio and Covid-19 outcome (life or death). But, our study showed that Adip/Lep ratio response is dependent of BMI and age, suggesting that this age and sex-specific changes should be considered when employing this potential marker for prognosis assessment of COVID-19. We also suggest that the described hormonal disruptions may impact lipoprotein profile. Two cohort studies (47, 48) demonstrated dysregulation of lipoprotein profile in COVID-19 patients, in particular, LDL-c and total cholesterol levels were reduced in patients with severe disease. These studies suggest a failure in liver metabolism control, due to a possible effect of the covid-related cytokine storm (47, 48). However, more studies are needed for better understand the involved mechanism.

Many factors may potentially lead to dysregulation of adipose tissue- endocrine function. We speculate that SARS-CoV-2 infection can affect the white adipose tissue *via* ACE2 receptors, impairing its endocrine function. Understanding the relationship between the adipose tissue and its hormones, and their relationship with BDNF, may facilitate the development of new therapeutic and immunometabolic strategies for COVID-19 in obese patients. The expanded WAT depots in obese subjects may also act as a storage site for the virus, favoring the rapid progression of SARS-CoV-2 infection, associated with increased virus shedding, immune activation, and cytokine production (49).

Shortcomings of the study include the fact that patients were admitted to the hospital already with a variety of metabolic disorders, and within different infection time spans. Therefore, it is important to establish whether COVID-19 induces these metabolic changes or aggravates previously existing ones. Another limitation is that the source of circulating BDNF was not investigated.

In summary, according to this prospective study, BDNF and BDNF/adiponectin ratio are possible markers of adverse clinical outcomes for COVID-19. BDNF levels were clearly influenced by BMI and age. In detail, as BMI increased, BDNF concentration decreased, a finding more significantly in individuals >60 years old. BDNF content was also lower in obese patients that required intensive care and in older patients who died. We propose the measurement of BDNF and adiponectin at hospital admission and during the progression of disease as to optimize treatment choices.

## Data Availability Statement

The raw data supporting the conclusions of this article will be made available by the authors, without undue reservation.

## Ethics Statement

The studies involving human participants were reviewed and approved by Human Research Ethics Committee of Pontificia Universidade Católica do Paraná (Number 31558020.8.0000.0103). The patients/participants provided their written informed consent to participate in this study.

## Author Contributions

LM, RP, and FL conceived the present idea. LM, EC, MD, FV, LM, and AG performed the experimental procedures. CB, AA, RP, and FL were responsible for the research funding and laboratory support. LM, MS, BS, and FL wrote the paper with input from all authors. TP and KK did a critical revision of the article. All authors read and approved the final manuscript.

## Funding

We thank the National Council of Technological and Scientific Development (CNPq)/Brazil, Coordination for the Improvement of Higher Education Personnel (CAPES)/Brazil, the São Paulo Research Foundation (FAPESP)/Brazil (Process Number, 2019/25626-26, Pontifícia Universidade Católica do Paraná (PUCPR)/Brazil and Banco Regional de Desenvolvimento do Extremo Sul (BRDE)/Brazil, Brazil for providing the financial support to conduct the research activities in the laboratory.

## Conflict of Interest

The authors declare that the research was conducted in the absence of any commercial or financial relationships that could be construed as a potential conflict of interest.

## Publisher's Note

All claims expressed in this article are solely those of the authors and do not necessarily represent those of their affiliated organizations, or those of the publisher, the editors and the reviewers. Any product that may be evaluated in this article, or claim that may be made by its manufacturer, is not guaranteed or endorsed by the publisher.
