# Supplementary material for: COVID-19 Outcome Relates With Circulating BDNF, According to Patient Adiposity and Age
Source: Front Nutr. 2021 Dec 10;8:784429. doi: 10.3389/fnut.2021.784429 (PMC8704131; doi:10.3389/fnut.2021.784429)
Supplement: Supplementary file 1 [file Data_Sheet_1.docx]

**Supplementary table 1.** Biochemical and metabolic profiles of patients with COVID-19 by sex and age.

|  | **Female**  **(n=59)** | **Male**  **(n=86)** | **P value** | **< 60 years**  **(n=93)** | ≥ **60 years**  **(n=52)** | **P value** |
| --- | --- | --- | --- | --- | --- | --- |
| Cholesterol, mg·dL^-1^ | 184.8 (165-217) | 182.6 (153-215) | .361 | 186.2 (157-216) | 179.6 (155-216) | .595 |
| Triglyceride, mg·dL^-1^ | 140.8 (111-190) | 133.6 (112-184) | .499 | 148.1 (113-187) | 126.0 (111-175) | .191 |
| HDL-c, mg·dL^-1^ | 30.3 (26-38) | 26.9 (24-32) | .003 | 28.0 (24-33) | 28.0 (24-33) | .980 |
| Non-HDL-c, mg·dL^-1^ | 154.5 (128-184) | 147.7 (122-189) | .823 | 155.0 (124-188) | 149.5 (124-186) | .657 |
| Insulin, μU·mL^-1^ | 30.4 (19-46) | 36.7 (22-46) | .272 | 31.4 (19-49) | 34.1 (23-44) | .936 |
| Glucose, mg·dL^-1^ | 89 (78-106) | 93.9 (82-113) | .251 | 90.0 (79-111) | 92.8 (81-109) | .990 |
| HOMA-IR | 6.6 (3-11) | 9.2 (5-12) | .178 | 7.9 (4-12) | 7.6 (5-12) | .898 |
| Cortisol nmol·L^-1^ | 7.0 (3-13) | 7.5 (4-40) | .493 | 7.0 (3-13) | 8.0 (4-49) | .205 |

Values are presented as median and interquartile range (Q1-Q3). Mean difference: Mann-Whitney test.

**Supplementary table 2**. Results of binary logistic regression analysis of predictors of COVID-19 outcome.

| **Variable** | **ICU** | | |  | **Death** | | |
| --- | --- | --- | --- | --- | --- | --- | --- |
|  | **OR** | **IC 95%** | **p-value** |  | **OR** | **IC 95%** | **p-value** |
| Age | 1.034 | 1.011; 1.057 | .003* |  | 1.099 | 1.050; 1.151 | .000** |
| Sex | .805 | .406; 1.597 | .805 |  | .571 | .190; 1.716 | .318 |
| BMI | 1.142 | 1.058; 1.232 | .001* |  | 1.047 | .949; 1.156 | .357 |
| BDNF | .985 | .975; .995 | .004* |  | .980 | .963; .998 | .034** |
| Adiponectin | 1.094 | .929; 1.287 | .283 |  | .929 | .715; 1.206 | .580 |
| Leptin | 1.004 | .986; 1.023 | .650 |  | .982 | .949; 1.016 | .297 |
| BDNF/Adip ratio | .953 | .922; .984 | .003* |  | .967 | .923; 1.013 | .163 |
| BDNF/Lep ratio | .971 | .905; 1.043 | .422 |  | 1.032 | .941; 1.131 | .504 |
| Adip/Lep ratio | .978 | .203; 4.715 | .978 |  | 6.416 | .925; 44.523 | .060 |
| Insulin | 1.000 | .983; 1.017 | .995 |  | .984 | .958; 1.010 | .226 |
| Glucose | 1.009 | .999; 1.018 | .067 |  | 1.000 | .986; 1.014 | .985 |
| HOMA-IR | 1.020 | .973; 1.070 | .406 |  | .984 | .911; 1.063 | .678 |
| Cholesterol | 1.006 | .998; 1.014 | .124 |  | 1.001 | .990; 1.013 | .836 |
| Triglycerides | 1.005 | .999; 1.011 | .094 |  | .988 | .976; 1.000 | .058 |
| HDL-c | .966 | .920; 1.014 | .158 |  | .916 | .838; 1.001 | .052 |
| Non-HDL-c | 1.007 | .999; 1.016 | .088 |  | 1.004 | .993; 1.016 | .491 |
| Cortisol | 1.016 | .984; 1.049 | .341 |  | 1.026 | .979; 1.075 | .288 |
| Hospitalization days | 1.240 | 1.154; 1.333 | .000* |  | 1.055 | 1.015; 1.097 | .006** |
| Diseases | .383 | .178; .824 | .014* |  | .104 | .013; .811 | .031** |
| Dyslipidemia | .506 | .199; 1.286 | .153 |  | .314 | .097; 1.023 | .054 |
| Hypertension | .391 | .194; .786 | .008* |  | .266 | .092; .770 | .015** |
| Diabetes | .503 | .229; 1.103 | .086 |  | .490 | .166; 1.445 | .196 |
| Respiratory | .464 | .235; .916 | .027* |  | .217 | .067; .704 | .011** |
| Others | .357 | .111; 1.153 | .085 |  | .705 | .142; 3.491 | .669 |
| Medication | .642 | .316; 1.305 | .221 |  | .091 | .012; .710 | .022** |

* Signifies associated variables with ICU admission.

** Signifies associated variables with outcome.

OR = odds ratio; IC = confidence interval.

**Supplementary table 3**. Results of multiple logistic regression analysis of predictors of COVID-19 outcome.

| Variable | ICU | | |  | Death | | |
| --- | --- | --- | --- | --- | --- | --- | --- |
|  | OR | IC 95% | p-value |  | OR | IC 95% | p-value |
| BDNF | .987 | .977; .998 | .018* |  | .985 | .966; 1.005 | .146 |
| Age | 1.038 | 1.011; 1.065 | .005* |  | 1.098 | 1.048; 1.151 | .000** |
| BMI | 1.173 | 1.073; 1.282 | .000* |  | 1.075 | .953; 1.213 | .239 |
| Sex | .779 | .352; 1.727 | .539 |  | .407 | .114; 1.450 | .165 |
|  |  |  |  |  |  |  |  |
| BDNF/Adip ratio | .949 | .916; .984 | .004* |  | 1.048 | .997; 1.101 | .065 |
| Age | 1.042 | 1.015; 1.070 | .002* |  | 1.102 | 1.049; 1.157 | .000** |
| BMI | 1.179 | 1.075; 1.293 | .000* |  | 1.042 | .917; 1.184 | .528 |
| Sex | .949 | .415; 2.170 | .901 |  | .389 | .109; 1.397 | .148 |
|  |  |  |  |  |  |  |  |
| Hospitalization | 1.230 | 1.138; 1.330 | .000* |  | .539 | .055; 5.273 | .595 |
| Age | 1.021 | .989; 1.053 | .199 |  | 1.097 | 1.043; 1.155 | .000** |
| BMI | 1.169 | 1.053; 1.297 | .003* |  | 1.082 | .958; 1.222 | .203 |
| Sex | .863 | .337; 2.208 | .758 |  | .414 | .120; 1.433 | .164 |
|  |  |  |  |  |  |  |  |
| Diseases | .805 | .318; 2.039 | .647 |  | .313 | .035; 2.827 | .301 |
| Age | 1.040 | 1.011; 1.070 | .006* |  | 1.097 | 1.040; 1.148 | .000** |
| BMI | 1.169 | 1.073; 1.275 | .000* |  | 1.075 | .953; 1.213 | .239 |
| Sex | .696 | .325; 1.490 | .351 |  | .405 | .117; 1.405 | .155 |
|  |  |  |  |  |  |  |  |
| Hypertension | .768 | .338; 1.743 | .528 |  | .707 | .197; 2.535 | .595 |
| Age | 1.040 | 1.012; 1.069 | .004* |  | 1.100 | 1.047; 1.155 | .000** |
| BMI | 1.166 | 1.069; 1.272 | .001* |  | 1.079 | .952; 1.223 | .236 |
| Sex | .687 | .320; 1.475 | .336 |  | .393 | .112; 1.384 | .146 |
|  |  |  |  |  |  |  |  |
| Respiratory | .717 | .318; 1.515 | .422 |  | .612 | .152; 2.464 | .489 |
| Age | 1.039 | 1.011; 1.068 | .005* |  | 1.096 | 1.042; 1.153 | .000** |
| BMI | 1.169 | 1.074; 1.273 | .000* |  | 1.080 | .956; 1.220 | .214 |
| Sex | .661 | .305; 1.435 | .295 |  | .391 | .111; 1.374 | .143 |
|  |  |  |  |  |  |  |  |

Adjusted for age, BMI and sex.

* Signifies associated variables with ICU admission.

** Signifies associated variables with outcome.

OR = odds ratio; IC = confidence interval.

**Supplementary table 4**. Results of multiple logistic regression analysis of predictors of COVID-19 outcome.

| Variable | UTI | | |  | Death | | |
| --- | --- | --- | --- | --- | --- | --- | --- |
|  | OR | IC 95% | p-value |  | OR | IC 95% | p-value |
| Hypertension | 2.254 | 1.095; 4.657 | 0.028* |  | 3.283 | 1.114; 9.675 | .031 |
| BDNF | .986 | .976; .996 | .008* |  | .982 | .963; 1.000 | .052 |
|  |  |  |  |  |  |  |  |
| Respiratory | .466 | .228; .951 | .036* |  | .216 | .065; .714 | .012 |
| BDNF | .985 | .975; .995 | .004* |  | .980 | .962; .998 | .030 |
|  |  |  |  |  |  |  |  |
| Hypertension | 2.112 | 1.005; 4.440 | .049* |  | 3.173 | 1.081; 9.318 | .036 |
| BDNF/Adip ratio | .956 | .925; .988 | .007* |  | .973 | .927; 1.022 | .275 |
|  |  |  |  |  |  |  |  |
| Respiratory | .484 | .233; 1.007 | .052* |  | .221 | ,067; .723 | .013 |
| BDNF/Adip ratio | .952 | .922; .984 | .003* |  | .968 | ,924; 1.014 | .170 |
|  |  |  |  |  |  |  |  |

* Signifies associated variables with ICU admission.

** Signifies associated variables with outcome.

OR = odds ratio; IC = confidence interval.

**Supplementary table 5**. Results of multiple logistic regression analysis of predictors of COVID-19 outcome.

| Variable | UTI | | |  | Death | | |
| --- | --- | --- | --- | --- | --- | --- | --- |
|  | OR | IC 95% | p-value |  | OR | IC 95% | p-value |
| Hypertension | 1.207 | .519; 2.805 | .662 |  | 1.234 | .350; 4.347 | .743 |
| BDNF | .987 | .976; .998 | .017* |  | .986 | .967; 1.005 | .144 |
| Age | 1.034 | 1.006; 1.063 | .016* |  | 1.091 | 1.039; 1.147 | .001** |
| BMI | 1.166 | 1.064; 1.277 | .001 |  | 1.058 | .941; 1.189 | .345 |
|  |  |  |  |  |  |  |  |
| Respiratory | .714 | .310; 1.643 | .428 |  | .696 | .172; 2.818 | .612 |
| BDNF | .987 | .976; .997 | .014* |  | .986 | .967; 1.005 | .145 |
| Age | 1.032 | 1.004; 1.061 | .027* |  | 1.088 | 1.033; 1.145 | .001** |
| BMI | 1.168 | 1.068; 1.276 | .001* |  | 1.057 | .943; 1.186 | .342 |
|  |  |  |  |  |  |  |  |
| Hypertension | 1.069 | .451; 2.535 | .879 |  | 1.123 | .320; 3.940 | .857 |
| BDNF/Adip ratio | .949 | .916; .983 | .004* |  | .976 | .927; 1.028 | .356 |
| Age | 1.041 | 1.012; 1.071 | .005* |  | 1.095 | 1.043; 1.050 | .000** |
| BMI | 1.177 | 1.071; 1.294 | .001* |  | 1.073 | .952; 1.209 | .251 |
|  |  |  |  |  |  |  |  |
| Respiratory | .859 | .353; 2.091 | .739 |  | .794 | .193; 3.275 | .750 |
| BDNF/Adip ratio | .949 | .916; .983 | .004* |  | .977 | .927; 1.028 | .370 |
| Age | 1.040 | 1.009; 1.071 | .010* |  | 1.093 | 1.037; 1.151 | .001** |
| BMI | 1.177 | 1.073; 1.291 | .001* |  | 1.072 | ,953; 1.206 | .246 |
|  |  |  |  |  |  |  |  |

* Signifies associated variables with ICU admission.

** Signifies associated variables with outcome.

OR = odds ratio; IC = confidence interval.
